# Impact of the Covid-19 pandemic on the management of gynecologic cancer: a Spanish survey. Observational, multicenter study

**DOI:** 10.1186/s12905-023-02633-4

**Published:** 2023-09-14

**Authors:** Myriam Gracia, Elena Rodríguez, María Dolores Diestro, Emanuela Spagnolo, Virginia García, Jaime Siegrist, Yolanda Pérez, Ignacio Zapardiel, Alicia Hernández

**Affiliations:** 1 Department of Gynecologic Oncology, La Paz University Hospital, Madrid, Spain; 2 Department of Gynecology, La Paz University Hospital Hospital Universitario La Paz, Madrid, Spain 28046 Paseo de la Castellana, 261,

**Keywords:** National survey, COVID-19 Infection, Gynecologic Oncology surgeons

## Abstract

**Background:**

The SARS-CoV-2 (Severe Acute Respiratory Syndrome Coronavirus 2) pandemic changed the distribution of healthcare resources, leading in many cases to the suspension of all non-essential treatments and procedures and representing a challenge for medical professionals. The objective of this study was to evaluate whether clinical protocols in gynecologic oncology care were modified as a result of the pandemic and to assess surgeons’ perceptions regarding the management of gynecologic cancers".

**Methods:**

Data were collected through an anonymous and voluntary survey sent via email to healthcare professionals in the field of gynecologic oncology in Spain.

**Results:**

A total of 75 gynecologic oncologists completed the online survey. Of these, 93.2% (69) reported working in public hospitals and 62.5% (45) in tertiary care hospitals. 97.3% (71) were affiliated with hospitals treating patients infected with SARS-CoV-2. 85.1% (63) of the respondents expressed concern about the SARS-CoV-2 pandemic and 52.1% (38) indicated that the pandemic impacted the diagnostic and therapeutic quality of care for oncology patients. SARS-CoV-2 nasopharyngeal swab PCR (Polymerase Chain Reaction) testing was always performed before surgical interventions by 97.3% (71), being considered a best practice in triage by 94.4% (68). 87.5% (63) reported no change in the type of surgical approach during the pandemic. 62.5% (45) experienced limitations in accessing special personal protective equipment for SARS-CoV-2. An impact on the follow-up of patients with gynecologic cancers due to the pandemic was reported by 70.4% (50).

**Conclusions:**

Most of the Spanish gynecologic oncologists who responded to our survey reported that the SARS-CoV-2 pandemic had affected their clinical practice. The primary measures implemented were an increase in telemedicine, restricting outpatient visits to high-risk or symptomatic patients and the use of SARS-CoV-2 screening prior to surgery. No major changes in the surgical approach or management of the treatment of ovarian, endometrial or cervical cancer during the pandemic were reported.

**Supplementary Information:**

The online version contains supplementary material available at 10.1186/s12905-023-02633-4.

## Background

The SARS-CoV-2 (Severe Acute Respiratory Syndrome Coronavirus 2) pandemic declared by the WHO (World Health Organization) on March 11, 2020 brought about a change in the distribution of healthcare resources, leading in many cases to the suspension of all non-essential treatments and procedures, especially during its peaks of highest incidence [1]. This situation represented a challenge for medical professionals, who had to decide which diagnostic and therapeutic approaches could be delayed and which could not. Such decisions were especially difficult in the case of oncology patients [1–3], a population considered to be at higher risk of SARS-CoV-2 infection due to their immunosuppressed state, secondary to the disease itself and many of the treatments used [1, 4, 5], as well as their age and the high prevalence of comorbidities [6, 7]. In addition, major surgery, as many of those performed on patients with gynecologic cancers involve, induces a suppression of the cellular immune response, which may be an additional risk factor for SARS-CoV-2 infection [5]. When making decisions in these patients, it is therefore of particular relevance to assess not only the therapeutic benefit but also the risk of exposure to SARS-CoV-2 [3]. Although histologic type, tumor stage, and patient characteristics determine the priority of surgical procedures, in recent years, risk of SARS-CoV-2 infection has become an additional factor in the decision-making process.

Spain was among the European countries most affected by SARS-CoV-2 during the first wave of the pandemic. In March 2020, it was the country with the second highest number of confirmed cases of COVID-19 in Europe. By the end of April, more than 61,000 people had been infected, of whom approximately 8,100 died [5]. The dramatic increase in the number of cases of the virus had a profound impact on hospitals, most of which were overcrowded with SARS-CoV-2 patients. This required the adoption of urgent measures to ensure that oncology patients received appropriate care. The number of surgeries were reduced, opting for non-surgical treatments in many cases, and patients were referred to other COVID-free oncology centers if the delay in surgery could affect their survival.

Such changes not only represented a challenge for healthcare professionals but also caused emotional stress for oncology patients, leading to higher rates of anxiety and depression compared to patients who did not undergo modifications to their treatment plan [4]. In addition, higher incidence of insomnia, asthenia and loss of appetite has been reported in these patients in relation to levels reported in the same patients before the pandemic [1].

The objective of this study was to evaluate whether clinical protocols in gynecologic oncology care were modified during the SARS-CoV-2 pandemic and to assess surgeons’ perceptions regarding the management of gynecologic cancers.

## Methods

This is an observational multicenter study. Data were collected through an anonymous survey completed voluntarily by healthcare professionals in the field of gynecologic oncology in participating centers in Spain between June 15–30, 2021. The online survey was sent by email to all members of the SEGO (Spanish Society of Gynecology and Obstetrics).

The survey consisted of 35 questions (Supplementary Material 1). The first seven questions collected data on the healthcare professionals responding to the survey (age, city and years in practice) and the center with which they are affiliated (job position, type of institution, presence of a gynecologic oncology unit, number of oncology patients per year). The following six questions asked about their level of concern about the pandemic and its impact on their daily practice, both on the quality of diagnostic and therapeutic procedures and the quality of care provided to patients with different types of gynecologic cancers. The next section of the survey involved five questions about pre-operative triage methods used and their opinion about them. This was followed by a set of nine questions regarding changes in surgical approach and SARS-CoV-2 protection measures used in the operating room. The last two sections consisted of four questions each and dealt with the changes observed in the management of different gynecologic cancers (ovarian, cervical and endometrial) during the pandemic, as well as the possible impact to patients.

### Statistical analysis

Quantitative data are presented as mean and standard deviation and qualitative variables as absolute values and relative frequencies (%). Analyses were performed using SPSS (Statistical Package for the Social Sciences) v.24 (IBM, Armonk, NY, USA).

## Results

### Sociodemographic characteristics

**Fig. 1:**
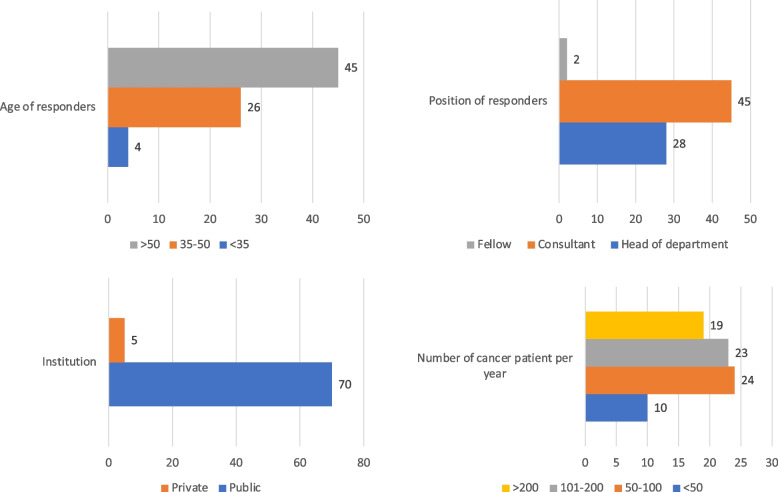
Sociodemographic characteristics of the 75 gynecologic oncologists from Spanish hospitals who completed the online survey

A total of 75 medical professionals specializing in gynecologic oncology from different hospitals in Spain completed the online survey. Of these, 45 (60%) were attending physicians, 28 (37.3%) were department heads and two (2.6%) were fellows. The mean age of the respondents was 47.82 ± 9.22 years and mean years in practice was 23.09 ± 9.89. Regarding the level of hospital care, 48 (64%) worked in tertiary care hospitals and 27 (37.5%) in first- or second-level hospitals. Sixty-seven (91.8%) reported having a gynecologic oncology unit in their workplace, while six (8.2%) did not have this type of unit. The primary sociodemographic characteristics may be observed in Fig. 1.

### Impact of the pandemic on the practice of gynecologic oncology

In terms of number of patients with gynecologic cancer managed annually at their center, 10 (13.5%) reported managing less than 50 patients per year, 24 (32.4%) reported managing between 50–100 patients, 23 (31.1%) between 101–200, and 19 (25.7%) managed more than 200 patients with gynecologic cancer per year. Seventy-one (97.3%) were affiliated with hospitals treating patients infected with SARS-CoV-2, while three (4.1%) worked in SARS-CoV-2-free centers.

A total of 63 (85.1%) respondents were concerned about the SARS-CoV-2 pandemic, while 11 (14.9%) did not express concern. Regarding the impact of the pandemic on their clinical practice, 59 (79.7%) reported changes due to the pandemic, while 16 (21.6%) reported no pandemic-related changes to their daily practice. A total of 38 (52.1%) participants indicated that the pandemic had impacted the diagnostic and therapeutic quality of care for oncology patients, compared to 35 (47.9%) who stated that the quality of care had not been impacted.

Asked about the impact of the pandemic on the quality of care received by patients with different types of gynecologic cancers, in cases of ovarian cancer, 48 (65.8%) of the respondents indicated that their clinical practices were unchanged compared to 26 (35.6%) who reported modifications. In endometrial cancer management, 48 (65.8%) reported no changes while 25 (34.2%) reported changes. Finally, 47 (64.4%) reported no change in the quality of care of patients with cervical cancer, compared to 26 (35.6%) reporting changes.

### Pre-operative SARS-Cov 2 screening

In terms of the measures adopted as triage for SARS-CoV-2 in patients undergoing surgery, that most frequently applied was SARS-CoV-2 nasopharyngeal swab PCR, with 69 (95.8%) of respondents reporting use of this method. Among other measures taken, 28 (38.9%) reported use of anamnesis (compatible symptomatology, close contact with SARS-CoV-2 positive patient), 14 (19.4%) used blood tests (hemogram, C-reactive protein), 12 (16.7%) performed antibody tests for SARS-CoV-2, 11 (15.3%) used rapid antigen tests, 10 (13.9%) chest X-rays and four (4.6%) chest CT scans. None of the respondents reported not performing any SARS-CoV-2 triage prior to surgery.

Seventy-one (97.3%) indicated that SARS-CoV-2 nasopharyngeal swab PCR was always performed before surgical interventions, three (4.1%) and one (1.4%) only in those cases where clinical or analytical signs of infection were present, respectively. None of the respondents indicated not performing SARS-CoV-2 PCR in any case.

Seventy-one (97.3%) of the professionals surveyed found the triage measures implemented to be effective, compared to two (2.7%) who did not. Regarding which triage method they considered best, 68 (94.4%) indicated SARS-CoV-2 nasopharyngeal swab PCR, 18 (25%) anamnesis, 14 (19.4%) rapid antigen testing, one (1.4%) chest X-ray and one (1.4%) blood tests. No one indicated chest CT as the best triage method.

### Surgeons’ perception of the management of SARS-CoV-2 during surgery

Thirty-seven (50%) respondents indicated that they felt at increased risk of SARS-CoV-2 infection during the course of their work, while the other 37 (50%) did not perceive an increased risk.

With respect to the degree of concern about the risk of SARS-CoV-2 infection by type of surgical approach, 64 (88.9%) participants did not feel greater concern with any approach in particular, seven (9.7%) expressed greater concern with open surgery, six (8.3%) with laparoscopic surgery and three (4.2%) with vaginal surgery. No one reported concern about the possibility of SARS-CoV-2 infection during robotic surgery.

Thirteen (18.3%) considered laparoscopy to involve an increased risk of SARS-CoV-2 infection, compared to 58 (81.7%) who did not perceive an increased risk.

A total of 63 (87.5%) participants reported no change in the type of surgical approach during the pandemic. Five (6.9%) reported a decrease in the laparoscopic approach in favor of open surgery, one (1.4%) a decrease in laparoscopy in favor of vaginal surgery, and three (4.2%) an increase in laparoscopy.

Forty (55.6%) respondents reported having adopted special personal protective measures in open surgery, while 33 (45.8%) did not report having adopted any additional protective measures. Conversely, in laparoscopic or robotic surgery, 40 (56.3%) stated that they had not adopted any SARS-CoV-2-specific protective measure, while 32 (45.1%) stated that they had. Specific protective measures adopted by type of surgical approach are described in Table 1.

**Table 1:** Specific protective measures adopted by type of surgical approach

	**Laparotomic surgery**	**Minimally Invasive Surgery**
FFP2 masks	52 (72.2)	40 (56.3)
Goggles protection	21 (29.2)	13 (18.3)
Screen protection	5 (6.9)	3 (4.2)
Usual protection (mask, gloves, gown)	45 (62.5)	42 (59.2)

Data are given in number (percentage)

Finally, regarding the accessibility of special personal protective equipment for SARS-CoV-2, 45 (62.5%) indicated that they had experienced limitations accessing it, compared to 27 (37.5%) who had experienced no difficulty.

### Management of ovarian cancer during the SARS-CoV-2 pandemic

In terms of changes observed in the management of this type of gynecologic cancer, 45 (63.4%) of the respondents reported no changes in primary cytoreductive surgeries, 24 (33.8%) reported a decrease in this type of intervention during the pandemic, and three (4.2%) an increase. Regarding the use of neoadjuvant chemotherapy in patients with ovarian cancer, 44 (63.8%) reported no change during the pandemic, 22 (31.9%) indicated an increase in use and three (4.3%) a decrease. Specific treatment options for advanced ovarian cancer are presented in Table 2.

**Table 2:** Specific treatment options for advanced ovarian cancer with surgically resectable advanced ovarian cancer

Diagnostic laparoscopy followed by PCR	22 (31.4)
PCR by laparotomic approach	20 (28.6)
Diagnostic laparoscopy followed by NAC	18 (25.7)
Diagnostic laparoscopy followed by PCR in the same surgical procedure	15 (21.4)
Radio guided biopsy followed by NAC	11 (15.7)

Data are given in number (percentage)

*NAC* Neoadjuvant chemotherapy

*PCR* Primary cytoreduction

Asked about changes in the treatment of ovarian cancer patients adopted during the pandemic, 49 (70%) reported no changes, 18 (25.7%) reported an increased use of neoadjuvant chemotherapy in order to defer surgery, and three (4.3%) reported avoiding surgery during the pandemic. None reported deferring chemotherapy.

### Management of cervical and endometrial cancer during the SARS-CoV-2 pandemic

Fifty-seven (80.3%) respondents reported no changes in the surgical management of endometrial cancer during the pandemic, while 56 (78.9%) reported no changes in the treatment of patients with cervical cancer. The changes to endometrial and cervical cancer management during the pandemic may be observed in Table 3.

**Table 3:** Changes in endometrial or cervical cancer management during pandemic outbreak

	**Endometrial Cancer**	**Cervical Cancer**
Delay of all treatments	11 (15.5)	8 (11.3)
Avoidance of surgical interventions in favor of increased use of systemic treatment	4 (5.6)	-
Avoidance of surgical interventions in favor radiotherapy	1 (1.4)	4 (5.6)
Avoiding lymph node staging	2 (2.8)	-
Avoiding lymphadenectomy in favor of sentinel lymph node biopsy	-	3 (4.2)

Data are given in number (percentage)

Thirty (42.3%) respondents reported no changes in follow-up of patients with gynecologic cancers as a consequence of the pandemic, 31 (43.7%) indicated a deferral of outpatient visits and 38 (53.5%) a greater use of telemedicine. A total of 19 (26.8%) respondents indicated having restricted follow-up to high-risk patients and 21 (29.6%) to symptomatic patients.

Finally, when asked about their opinion of the impact that the pandemic could have on the follow-up of patients with gynecologic cancers, 50 (70.4%) reported some impact, while 21 (29.6%) did not believe it would have an impact.

## Discussion

Our study is the first to evaluate the impact of the SARS-CoV-2 pandemic on the management of patients with gynecologic cancer in Spain through a national survey. In it, we reflect the reality of the changes produced following lockdown and the first year of the pandemic. Most respondents held a position as attending physician in the public health system which is highly representative of the reality of the country.

A total of 79.7% of the Spanish gynecologic oncologists who responded to our survey reported that SARS-CoV-2 pandemic had affected their daily clinical practice. Similar results were found by other authors. A higher percentage—97.3%— of physicians forced to change their customary practices due to the pandemic was reported in a recent study by Martinelli et al., but this article reflects perceptions during the first year of the outbreak [2] while our study was conducted one year later. Diagnostic and therapeutic quality were also affected by the pandemic in the view of 52.1% of our respondents. Stratifying by type of cancer, quality of care was impacted in ovarian, endometrial and cervical cancers.

Assessment of the pre-operative triage measures adopted for COVID 19 is a relatively simple indicator to measure and the responses in this regard were highly homogeneous. In our survey, 95.8% of the respondents used SARS-CoV-2 nasopharyngeal swab PCR as a pre-operative triage method and only 13.9% used chest X-rays. In contrast, professionals in other countries reported that while patients’ COVID-19 status before surgery was evaluated primarily with COVID-19 nasopharyngeal swabs (53.7%), radiological assessments (chest X-ray 41.5% and chest CT scan 30.5%) were also widely implemented [2].

The European Society for Medical Oncology (ESMO) guidelines also advocate a nasopharyngeal swab for SARS-CoV-2 prior to surgery and recommend postponing surgery in cases with a positive result in order to minimize the risk of postsurgical complications and the exposure of healthcare professionals [6, 7].

Half of the respondents to our survey indicated that they felt at increased risk of SARS-CoV-2 infection during their clinical practice. This concern seems to be well-founded; in fact, other authors have described infection rates ranging from 5.62% among healthcare workers in Southwest Iran to 9.1% in a hospital in Spain during the first year of the pandemic [5, 8]. In addition, 62.5% of the respondents indicated that they had experienced limitations in accessing special personal protective equipment for SARS-CoV-2 since the pandemic began.

The surgical approach has also been studied to assess whether this influences the spread of COVID-19. Specifically, the laparoscopic approach has been questioned due to the possibility of greater exposure to the virus with the use of pneumoperitoneum [9]. Although the presence of other pathogens (human papillomavirus, hepatitis B virus, human immunodeficiency virus and Corynebacterium) in laparoscopic procedures has been well documented by other studies, evidence on the COVID-19 specific risk in laparoscopic surgeries was limited at the beginning of the pandemic [10]. Today there is no reliable evidence to suggest a risk of COVID-19 transmission via laparoscopic pneumoperitoneum and surgical smoke [11]. In our study, most respondents (88.9%) did not indicate greater concern about any particular approach, which is consistent with the fact that 87.5% did not change the type of surgical approach during the pandemic.

Regarding special personal protective measures, the use of FFP2 masks was the measure implemented most frequently both in open surgery and laparoscopic and robotic surgery (72.2% and 73.2%, respectively), following current recommendations provided by the literature [12].

In response to our questions about the changes adopted in the management of each type of gynecologic cancer, we observed a slight decrease in primary cytoreductive surgeries and an increase in favor of neoadjuvant chemotherapy for ovarian cancer, reported by 33.8% and 31.9% of the respondents, respectively. Bogani et al. also found in a decrease of approximately 20–25% in radical surgical procedures for ovarian cancer in Italy [1]. Similarly, Martinelli et al. reported a decrease in staging surgeries in advanced tumors, increased use of neoadjuvant chemotherapy and postponement of interval surgeries [2]. This reduction in the number of radical surgeries represented a decrease in the need for patient admissions to Intensive Care Units, which were overcrowded with patients infected with SARS-CoV-2.

A minority of respondents (4.3%) indicated having avoided ovarian cancer surgery during the pandemic, although none deferred the use of chemotherapy. In terms of cervical and endometrial cancer management, most respondents (80.3%) reported no changes, although some indicated having avoided surgery in favor of systemic treatment or radiotherapy. This approach is consistent with responses and guidelines from other countries proposing the use of hypofractionated radiotherapy or oral chemotherapy drugs [2].

Finally, most respondents indicated an impact on the follow-up of these patients due to the pandemic (70.4%). Among the primary measures adopted we found an increased use of telemedicine or restricting outpatient visits to high-risk or symptomatic patients, measures that have also been reported in previous literature [2–4]. Although some studies have found that gynecologic cancer patients were highly satisfied with the use of telemedicine during the SARS-CoV-2 pandemic [13, 14], to date no prospective randomized study with follow-up comparing telemedicine to on-site visits in terms of patient quality of life and recurrence rate has been published [15].

The most significant limitation to our study is that COVID-19 is constantly changing and some of the practices and measures we describe may have changed over the course of the pandemic. On the other hand, as a strength of our study, it should be noted that all the measures detailed could be adopted for other hypothetical health emergencies. Based on the lessons learned during the COVID-19 pandemic, collaborative studies should be conducted to identify best practices in triage and treatment options for gynecologic oncology patients to confront pandemics in the future.

## Conclusions

Most of the Spanish gynecologic oncologists who responded to our survey reported that the COVID-19 pandemic affected their daily clinical practice, with more than half indicating that the diagnostic and therapeutic quality of care was impacted. The primary measures implemented were an increase in telemedicine, restricting outpatient visits to high-risk or symptomatic patients and the use of SARS-CoV-2 screening prior to surgery, specifically nasopharyngeal swab PCR. The majority of the respondents indicated no changes in the surgical approach or management of ovarian, endometrial or cervical cancer during the pandemic.

The creation of multidisciplinary teams, of particular relevance in the treatment of oncology patients, has become an essential part of the decision-making process during the SARS-CoV-2 pandemic. New technologies have made this possible, providing the means for healthcare professionals to stay in contact at a time when social interactions were highly restricted in order to control the incidence of the virus.

### Supplementary Information

**Additional file 1.**

## Data Availability

The datasets used and analyzed during the current study are available from the corresponding author on reasonable request.

## Abbreviations

WHO: World Health Organization
SEGO: Spanish Society of Gynecology and Obstetrics
CT: Computed tomography
ESMO: European Society for Medical Oncology

## References

[1] Bogani G, Apolone G, Ditto A (2020). Impact of COVID-19 in gynecologic oncology: a Nationwide Italian Survey of the SIGO and MITO groups. J Gynecol Oncol.

[2] Martinelli F, Garbi A (2020). Change in practice in gynecologic oncology during the COVID-19 pandemic: a social media survey. Int J Gynecol Cancer.

[3] Ramirez PT, Chiva L, Eriksson AGZ (2020). COVID-19 Global Pandemic: Options for Management of Gynecologic Cancers. Int J Gynecol Cancer.

[4] Kirby A, Drummond FJ, Lawlor A, Murphy A (2022). Counting the social, psychological, and economic costs of COVID-19 for cancer patients. Support Care Cancer.

[5] de Santiago J, Yelo C, F Chereguini M (2020). COVID-19: gynecologic cancer surgery at a single center in Madrid. Int J Gynecol Cancer.

[6] Colombo I, Zaccarelli E, Del Grande M (2020). ESMO management and treatment adapted recommendations in the COVID-19 era: gynaecological malignancies. ESMO Open.

[7] Hwang ES, Balch CM, Balch GC (2020). Surgical Oncologists and the COVID-19 Pandemic: Guiding Cancer Patients Effectively through Turbulence and Change. Ann Surg Oncol.

[8] Sabetian G, Moghadami M, Hashemizadeh Fard Haghighi L (2021). COVID-19 infection among healthcare workers: a cross-sectional study in southwest Iran. Virol J.

[9] Zheng MH, Boni L, Fingerhut A (2020). Minimally Invasive Surgery and the Novel Coronavirus Outbreak: Lessons Learned in China and Italy. Ann Surg.

[10] Mallick R, Odejinmi F, Clark TJ (2020). Covid 19 pandemic and gynaecological laparoscopic surgery: knowns and unknowns. Facts Views Vis Obgyn.

[11] Antunes D, Lami M, Chukwudi A (2021). COVID-19 infection risk by open and laparoscopic surgical smoke: A systematic review of the literature. Surgeon.

[12] Chiofalo B, Baiocco E, Mancini E (2020). Practical recommendations for gynecologic surgery during the COVID-19 pandemic. Int J Gynaecol Obstet.

[13] Mojdehbakhsh RP, Mora Hurtado AC, Uppal S, Milakovich H, Spencer RJ (2022). The long game: Telemedicine patient satisfaction metrics and methods of recurrence detection for gynecologic cancer patients throughout the initial year of the COVID-19 pandemic. Gynecol Oncol Rep.

[14] Uppal A, Kothari AN, Scally CP, Roland CL, Bednarski BK, Katz MHG, Vauthey JN, Chang GJ, D3CODE Team (2022). Adoption of Telemedicine for Postoperative Follow-Up After Inpatient Cancer-Related Surgery. JCO Oncol Pract.

[15] Lin H, Ye M, Chan SW, Zhu J, He H (2020). The effectiveness of online interventions for patients with gynecological cancer: An integrative review. Gynecol Oncol.

