# Supplementary material for: Impact of the Covid-19 pandemic on the management of gynecologic cancer: a Spanish survey. Observational, multicenter study
Source: BMC Womens Health. 2023 Sep 14;23:488. doi: 10.1186/s12905-023-02633-4 (PMC10500789; doi:10.1186/s12905-023-02633-4)
Supplement: Supplementary file 1 — Additional file 1. [file 12905_2023_2633_MOESM1_ESM.docx]

**Impact of the Covid-19 pandemic on gynaecological cancer management: national survey. GineonCoVIDsurg study.**

Voluntary and anonymous survey.

**GYNAECOLOGICAL ONCOLOGY IN TIMES OF PANDEMIC COVID-19**

**GENERAL INFORMATION**

1. AGE:
2. CITY:
3. YEARS OF PROFESSIONAL DEDICATION:
4. JOB POSITION:
   1. Head of service
   2. Head of section
   3. Consultant
   4. Fellow
5. TYPE OF HEALTH INSTITUTION:
   1. Public Hospital
   2. Private Hospital
6. LEVEL OF HOSPITAL CARE:
   1. Tercer nivel/Centro de referencia
   2. First or second level.
7. DO YOU HAVE A GYNAECOLOGY ONCOLOGY UNIT IN YOUR HOSPITAL?
   1. YES
   2. NO
8. NUMBER OF GYNAECOLOGICAL CANCER PATIENTS PER YEAR:
   1. <50
   2. 50-100
   3. 101-200
   4. >200

**GYNAECOLOGICAL ONCOLOGY IN TIMES OF PANDEMIC COVID-19**

**CLINICAL PRACTICE IMPACT**

1. Are you concerned about the COVID-19 pandemic? (0- No; 1-Yes)
2. Is COVID-19 impacting your clinical practice? (0- No; 1-Yes)
3. Is COVID-19 influencing the quality of diagnosis and treatment of its patients? (0- No; 1-Yes)
4. Is COVID-19 influencing the quality of care of your ovarian cancer patients? (0- No; 1-Yes)
5. Is COVID-19 influencing the quality of care of your endometrial cancer patients? ((0- No; 1-Yes)
6. Is COVID-19 influencing the quality of care of your cervical cancer patients? (0- No; 1-Yes)

**PRESURGICAL COVID-19 SCREENING**

1. Your hospital is:
   1. COVID-19 free.
   2. Not COVID-19 free.
2. Is your hospital adopting COVID-19 screening measures for patients requiring surgery?
   1. None
   2. Medical history (fever, cough, positive patient contact.)
   3. Nasopharyngeal exudate (PCR COVID-19).
   4. Rapid antigen test.
   5. COVID-19 Antibody test.
   6. Blood test.
   7. Chest X-ray
   8. Thoracic CT scan.

18 ¿ When to perform nasopharyngeal exudate (PCR COVID-19) prior to surgery?

1. Medical history sugesting COVID-19 infection.
2. Radiological data suggestive of infection.
3. Blood test suggesting infection.
4. Always.
5. Never.
6. Do you believe triage measures are effective? (0- No; 1-Yes)
7. ¿ Personally, which one do you think the screening method?
   1. Medical history (fever, cough, positive patient contact.)
   2. Nasopharyngeal exudate (PCR COVID-19).
   3. Rapid antigen test.
   4. COVID-19 Antibody test.
   5. Blood test.
   6. Chest X-ray
   7. Thoracic CT scan.
   8. None.

**SURGERY AND PROTECTIVE MEASURES DURING THE COVID-19 PANDEMIC.**

1. Do you feel at risk of being infected by COVID-19 during the course of your work? (0- No; 1-Yes)
2. Are you concerned about performing any of these surgical approaches?
   1. Laparoscopy
   2. Robotic surgery
   3. Open surgery
   4. Vaginal surgery
   5. Not concerned
3. Do you believe laparoscopic surgery increases risk of infection.? (0- No; 1-Yes)
4. Ha surgical approach changed during the COVID -19 pandemic.?
   1. Laparoscopy has decreased in favour of open surgery.
   2. Laparoscopy has decreased in favour of vaginal surgery.
   3. Laparoscopy has decreased inf favour of robotic surgery.
   4. Laparoscopy has increased.
   5. No changes.
5. Are you adopting specific protection measures in open surgery? (0- No; 1-Yes)
6. What personal protective measures are you using during open surgery?

a) Usual equipment (mask, gloves, gown).

b) Protection with screen.

c) Protection with goggles

d) FFP2 mask.

1. Are you adopting specific protective measures in laparoscopic or robotic surgery? (0- No; 1-Yes)
2. Which personal protective measures are you using during laparoscopic or robotic surgery??

a) Usual equipment (mask, gloves, gown).

b) Protection with screen.

c) Protection with goggles

d) FFP2 mask.

1. There has been any limitation in personal protective equipment in your centre? (0- No; 1-Yes)

**OVARIAN CANCER MANAGEMENT**

1. Regarding primary cytoreduction surgery during the COVID-19 pandemic:

a) No change.

b) Decreased.

c) Increased.

1. Regarding neoadjuvant chemotherapy during COVID-19 pandemic:

a) No change.

b) Decreased.

c) Increased.

1. Treatment modality of choice for patients with advanced ovarian cancer (with potentially resectable disease) during the COVID-19 pandemic:
   - 1. Diagnostic laparoscopy followed by primary cytoreduction in the same surgical act.
     2. Diagnostic laparoscopy and primary cytoreduction in a second procedure.
     3. Primary debulking surgery by laparotomy.
     4. Diagnostic laparoscopy followed by neoadjuvant chemotherapy.
     5. Radio-guided biopsy followed by neoadjuvant chemotherapy.
2. Changes in management of patients with recurrent ovarian cancer during the COVID- 19 pandemic:

a) Avoiding surgery.

b) Avoiding chemotherapy.

c) Neoadjuvant chemotherapy to delay surgery.

d) No changes.

**ENDOMETRIAL AND CERVICAL CANCER MANAGEMENT**

1. Regarding the surgical treatment of endometrial cancer during the COVID-19 pandemic:

a) No changes.

b) Avoiding surgery (in favour of radiotherapy).

c) Avoiding surgery (in favour of systemic treatment).

d) Hysterectomy only (avoid lymph node staging).

e) Delaying all types of treatment.

1. Changes in cervical cancer management:
   1. Avoiding surgery (in favour of radiotherapy).
   2. Reducing radicality of hysterectomy.
   3. Avoiding lymphadenectomy in favor of sentinel node biopsy.
   4. Delaying all types of treatment.
   5. No changes.
2. Changes in follow up:

a) Follow-up only for high-risk patients.

b) Follow-up for symptomatic patients.

c) Telemedicine.

d) Postponement of visits.

e) No change.

1. Will the COVID-19 pandemic have an impact on the follow-up of your patients? (0- No; 1-Yes)
